# Genetic epidemiology of autoinflammatory disease variants in Indian population from 1029 whole genomes

**DOI:** 10.1186/s43141-021-00268-2

**Published:** 2021-12-14

**Authors:** Abhinav Jain, Rahul C. Bhoyar, Kavita Pandhare, Anushree Mishra, Disha Sharma, Mohamed Imran, Vigneshwar Senthivel, Mohit Kumar Divakar, Mercy Rophina, Bani Jolly, Arushi Batra, Sumit Sharma, Sanjay Siwach, Arun G. Jadhao, Nikhil V. Palande, Ganga Nath Jha, Nishat Ashrafi, Prashant Kumar Mishra, Vidhya A.K., Suman Jain, Debasis Dash, Nachimuthu Senthil Kumar, Andrew Vanlallawma, Ranjan Jyoti Sarma, Lalchhandama Chhakchhuak, Shantaraman Kalyanaraman, Radha Mahadevan, Sunitha Kandasamy, Pabitha B. M,, Raskin Erusan Rajagopal, Ezhil Ramya J., Nirmala Devi P., Anjali Bajaj, Vishu Gupta, Samatha Mathew, Sangam Goswami, Mohit Mangla, Savinitha Prakash, Kandarp Joshi, Meyakumla, Sreedevi S., Devarshi Gajjar, Ronibala Soraisham, Rohit Yadav, Yumnam Silla Devi, Aayush Gupta, Mitali Mukerji, Sivaprakash Ramalingam, Binukumar B. K., Vinod Scaria, Sridhar Sivasubbu

**Affiliations:** 1 CSIR-Institute of Genomics and Integrative Biology, New Delhi, 110025 India; 2 Academy of Scientific and Innovative Research (AcSIR), Ghaziabad, Uttar Pradesh 201002 India; 3 Department of Zoology, RTM Nagpur University, Nagpur, Maharashtra 440033 India; 4 Department of Zoology, Shri Mathuradas Mohota College of Science, Nagpur, Maharashtra 440009 India; 5 Department of Anthropology, Vinoba Bhave University, Hazaribag, Jharkhand 825301 India; 6 Department of Biotechnology, Vinoba Bhave University, Hazaribag, Jharkhand 825301 India; 7 Department of Biochemistry, Dr. Kongu Science and Art College, Erode, Tamil Nadu 638107 India; 8 Thalassemia and Sickle Cell Society, Hyderabad, Telangana 500052 India; 9 Department of Biotechnology, Mizoram University, Aizawl, Mizoram 796004 India; 10 Department of Pathology, Civil Hospital Aizawl, Mizoram, 796001 India; 11 TVMC, Tirunelveli Medical College, Tirunelveli, Tamil Nadu 627011 India; 12 Department of Microbiology, St.Pious X Degree & PG College for Women, Hyderabad, Telangana 500076 India; 13 Department of Microbiology, The Maharaja Sayajirao University of Baroda, Vadodara, Gujarat 390002 India; 14 Department of Dermatology, Venereology and Leprology, Regional Institute of Medical Sciences, Imphal, Manipur 795004 India; 15 CSIR- North East Institute of Science and Technology, Jorhat, Assam 785006 India; 16 Department of Dermatology, Dr. D.Y. Patil Medical College, Pune, Maharashtra 411018 India

**Keywords:** Autoinflammatory disorder, Genetic epidemiology, American College of Medical Genetics and Genomics, Allele frequency, Haplotype ancestry

## Abstract

**Background:**

Autoinflammatory disorders are the group of inherited inflammatory disorders caused due to the genetic defect in the genes that regulates innate immune systems. These have been clinically characterized based on the duration and occurrence of unprovoked fever, skin rash, and patient’s ancestry. There are several autoinflammatory disorders that are found to be prevalent in a specific population and whose disease genetic epidemiology within the population has been well understood. However, India has a limited number of genetic studies reported for autoinflammatory disorders till date. The whole genome sequencing and analysis of 1029 Indian individuals performed under the IndiGen project persuaded us to perform the genetic epidemiology of the autoinflammatory disorders in India.

**Results:**

We have systematically annotated the genetic variants of 56 genes implicated in autoinflammatory disorder. These genetic variants were reclassified into five categories (i.e., pathogenic, likely pathogenic, benign, likely benign, and variant of uncertain significance (VUS)) according to the American College of Medical Genetics and Association of Molecular pathology (ACMG-AMP) guidelines. Our analysis revealed 20 pathogenic and likely pathogenic variants with significant differences in the allele frequency compared with the global population. We also found six causal founder variants in the IndiGen dataset belonging to different ancestry. We have performed haplotype prediction analysis for founder mutations haplotype that reveals the admixture of the South Asian population with other populations. The cumulative carrier frequency of the autoinflammatory disorder in India was found to be 3.5% which is much higher than reported.

**Conclusion:**

With such frequency in the Indian population, there is a great need for awareness among clinicians as well as the general public regarding the autoinflammatory disorder. To the best of our knowledge, this is the first and most comprehensive population scale genetic epidemiological study being reported from India.

**Supplementary Information:**

The online version contains supplementary material available at 10.1186/s43141-021-00268-2.

## Background

Autoinflammatory disorders are the growing group of Mendelian disorders caused by genetic defects in the genes which regulate the innate immune system. These disorders have been characterized by recurrent episodes of fever, abdominal pain, skin rashes, arthritis, serositis, conjunctivitis, or cutaneous signs that lack specificity and therefore make diagnosis difficult [1, 2]. These disorders are clinically diagnosed based on the age of onset, duration of fever and flares, type of rash, family history, and patient’s ancestry [3]. Autoinflammatory disorders have a strong genetic background, multiple new genes or variants have been recently discovered [4–7]. The recent advancement in next-generation sequencing has resulted in the identification of more than 30 new genes associated with autoinflammatory disorders [8, 9]. Classically, most monogenic autoinflammatory disorders follow autosomal dominant and autosomal recessive modes of inheritance. Molecular diagnosis along with clinical criteria is essential for confirmation of the disease as well as it aids in discovering new disease [10]. Even autoinflammatory disorders manifest heterogeneity in phenotype–genotype correlation (i.e., mutation in the same gene can have varying severity and clinical manifestation and mutation in different genes can result in similar clinical characteristics) [11–13].

Identification of the causal variants in the genes responsible for autoinflammatory disorder mainly uses clinical or whole exome sequencing. However, 60% of patients suspected with autoinflammatory disorder remain molecularly undiagnosed using these sequencing technologies [14]. The undiagnosis could be due to the inability of the sequencing platforms in calling structural variants (SVs) [15], missing non-coding variants [16] as well as inadequate coverage of the coding region [17], and high-quality SNVs [18]. However the whole genome sequencing (WGS) has the ability to identify variants in difficult-to-diagnose cases. Recently, our group has identified ~ 5 Kb deletion in the primary immunodeficiency disorder (PID) patients that could not be identified using whole exome sequencing [19]. Also, Thaventhiran et al. has identified eight SVs by performing the WGS of 1318 patients affected with PID, that could be missed using the WES [20]. WGS has also been implemented at the population scale for autoinflammatory disorders to comprehend the genetic epidemiology of the Qatari population [21]. Even though India is a hotspot for the genetic disorder due to the prevalent practice of endogamy and consanguineous marriages, migration, and large population size, the genetic epidemiology of autoinflammatory disorders in the country has not been  studied much except for a few reports [22].

The whole-genome sequence data for cosmopolitan Indian populations encompassing 1029 individuals as part of the IndiGen programme [23] motivated us to estimate the genetic epidemiology of autoinflammatory disorders. In the present analysis, we have performed extensive data mining as well as integrative analysis to evaluate the pathogenicity of the variant according to ACMG-AMP guidelines. We have also further analyzed the variant prevalence in India compared to the global population. This is the first most comprehensive genetic epidemiology performed for autoinflammatory disorders in India.

## Methods

### IndiGen dataset and variant annotation

A total of 59,646,267 genetic variants including single-nucleotide variants and Indels from the IndiGen dataset were considered for the analysis. The data was derived from whole genome sequencing of 1029 cosmopolitan healthy Indians with a well-written informed consent obtained [23]. These are self-declared healthy individuals selected from different states of India without any provisional diagnosis of autoinflammatory disorder. It includes 495 males and 534 females with mean ages of 41.35 and 32.96 years, respectively [23]. These variants obtained were annotated using a tool called ANNOVAR (v. 2018-04-06) [24]. ANNOVAR provides annotations from multiple databases that include RefGene [25] and dbSNP (avsnp150) [26] that provide detailed information  about the variant, dbNSFP35a that comprises data of multiple pathogenicity prediction tools [27]; global population databases (gnomAD V3 [28], 1000 Genome Project [29], Esp6500 [30], and Greater Middle East (GME)) provide allele frequency of different ancestry [31]. Finally, variant clinical significance of variants was was retrieved from ClinVar (ver 2020-01-13) database [32].

### IndiGen variant filtering

Out of the total IndiGen variants, we have extracted variants from 56 genes associated with the 47 autoinflammatory disorders. These autoinflammatory disorder genes were selected by the experts of the International Union of Immunological Societies (IUIS) [33] and Infever as tabulated in Table 1. Further, those variants that are mapped to the exonic (except synonymous) and splicing region or those that were pathogenic and likely pathogenic variants at the other genomic positions as per ClinVar were prioritized. Also, variants whose allele frequency is less than 0.05 in the global population datasets (1000 genome project, gnomAD V3, and Esp6500) were considered for further analysis. As the variants with allele frequency greater than 0.05 were considered polymorphic and present in the large number of healthy individuals across different populations.

**Table 1:** Genes associated with autoinflammatory disorder with their respective mode of inheritance

***Gene***	Disease	Mode of inheritance
*ACP5*	Spondyloenchondrodysplasia with immune dysregulation	AR
*ADA2*	Deficiency of adenosine deaminase 2 (DADA2)	AR
*ADAM17*	Inflammatory skin and bowel disease, neonatal	AR
*ADAR1; ADAR*	Aicardi-Goutières syndrome	AR
*ALPI*	Monogenic inflammatory bowel disease	AR
*AP1S3*	Psoriasis 15, pustular	AD
*CARD14*	Familial psoriasis/ CARD14-mediated pustular psoriasis (CAMPS/PSORS2)	AD
*CDC42*	Takenouchi-Kosaki syndrome	AD
*COPA*	Autoimmune interstitial lung, joint, and kidney disease	AD
*DNASE1L3*	Systemic lupus erythematosus	AR
*DNASE2*	Type I interferon–mediated autoinflammation	AD
*HAVCR2*	T cell lymphoma, subcutaneous panniculitis	AR
*IFIH1*	Aicardi-Goutières syndrome	AD
*IL10*	Interleukin 10 deficiency (IL10D)	AR
*IL10RA*	Inflammatory bowel disease 28 (IBD28)/ interleukin 10 receptor A deficiency (IL10R1D)	AR
*IL10RB*	Inflammatory bowel disease 25 (IBD25)/ interleukin 10 receptor B deficiency (IL10R2D)	AR
*IL1RN*	Deficiency of interleukin-1ß (IL-1ß) receptor antagonist/ osteomyelitis, sterile multifocal with periostitis pustulosis (DIRA/OMPP)	AR
*IL36RN*	Deficiency of interleukin-36-receptor antagonist/ generalized pustular psoriasis (GPP)/ (DITRA/PSORP)	AR
*LACC1*	Juvenile arthritis	AR
*LPIN2*	MAJEED/ chronic recurrent multifocal osteomyelitis, congenital dyserythropoietic anemia, & neutrophilic dermatosis	AR
*MEFV*	Familial Mediterranean fever	AR; AD
*MVK*	Hyperimmunoglobulinemia D with periodic fever syndrome (HIDS); melvanoic aciduria (MA)	AR
*NCSTN*	Acne inversa, familial	AD
*NLRC4*	Autoinflammation with infantile enterocolitis (AIFEC)	AD
*NLRP12*	NLRP12-associated periodic fever syndrome/ familial cold autoinflammatory syndrome 2, or Guadaloupe periodic fever (NLRP12/FCAS2)	AD
*NLRP1*	Autoinflammation with arthritis and dyskeratosis	AR; AD
*NLRP3*	Familial cold autoinflammatory syndrome (FCAS); Muckle-Wells syndrome (MWS); neonatal onset multisystemic inflammatory disorder/ chronic infantile neurological cutaneous articular syndrome (NOMID/CINCA)	AD
*NLRP7*	Hydatidiform mole, recurrent, 1 (HYDM1)	AR
*NOD2*	Juvenile systemic granulomatosis–Blau syndrome, pediatric granulomatous arthritis (PGA), Crohn's disease early onset sarcoidosis, or Jabs syndrome (BLAU/PGA/EOS)	AD
*OAS1*	Pulmonary alveolar proteinosis with hypogammaglobulinemia	AR
*OTULIN*	Otulipenia	AR
*PLCG2*	PLCG2-associated antibody deficiency and immune dysregulation/ familial atypical cold urticaria (FACU) (PLAID/FCAS3); autoinflammation and PLCG2-associated antibody deficiency and immune dysregulation (APLAID)	AD
*POLA1*	Type I interferon–mediated autoinflammation	XLR
*POMP*	Chronic atypical neutrophilic dermatosis with lipodystrophy and elevated temperature—Nakajo Nishimura syndrome (CANDLE/PRAAS)	AR
*PSMA3*		
*PSMB4*		
*PSMB8*		
*PSMB9*		
*PSMG2*	Chronic atypical neutrophilic dermatosis with lipodystrophy and elevated temperature (CANDLE)	AR;AD
*PSTPIP1*	Pyogenic sterile arthritis, pyoderma gangrenosum and acne syndrome (PAPA)	AD
*RBCK1*	Polyglucosan body myopathy, early-onset, with or without immunodeficiency (PBMEI)	AR
*RNASEH2A*	Aicardi-Goutières syndrome	AR
*RNASEH2B*		
*RNASEH2C*		
*SAMHD1*	Chilblain lupus	AD
*SH3BP2*	Cherubism	AD
*SLC29A3*	SLC29A3 spectrum disorder (SLC29A3)	AR
*TMEM173*	Sting-associated vasculopathy, infantile-onset (SAVI)	AD
*TNFAIP3*	Autoinflammatory syndrome, familial, Behcet-like (AISBL)	AD
*TNFRSF11A*	TNFRSF11A-associated hereditary fever disease (TRAPS11)	AD
*TNFRSF1A*	TNF receptor-associated periodic syndrome (TRAPS)	AD
*TREX1*	Systemic lupus erythematosus; Aicardi-Goutières syndrome; Chilblain lupus	AR; AD
*TRIM22*	Inflammatory bowel disease	AR
*TRNT1*	Sideroblastic anemia with B cell immunodeficiency, periodic fevers, and developmental delay	AR
*USP18*	Pseudo-TORCH syndrome	AR
*WDR1*	Autoinflammatory periodic fever, immunodeficiency, and thrombocytopenia (PFIT)	AR

*AR* autosomal recessive, *AD* autosomal dominant, *XLR* X-linked recessive

### Datasets of disease-associated genetic variants

We downloaded genetic variants from two well-annotated databases (i.e., ClinVar (ver 2020-01-13) [32] and Infevers [8, 34]). ClinVar is an up-to-date database composed of genetic variants in multiple gens associated with multiple disorders classified as pathogenic, likely pathogenic, benign, likely benign, variant of uncertain significance (VUS), or of conflicting evidence from different sources. We filtered variants that were pathogenic, likely pathogenic, VUS, and conflicting evidence as per ClinVar  and retrieved variants in 56 genes associated with 47 autoinflammatory disorders. The Infevers is a publicly available database that [35] has a total of 2112 genetic variants in 38 genes involved in 34 autoinflammatory disorders. The filtered genetic variants from ClinVar and Infever were merged and mapped to the IndiGen filtered variants.

### Classification of genetic variants according to ACMG and AMP guidelines

The American College of Medical Genetics and Genomics and the Association of Molecular Pathology (ACMG-AMP) experts have provided comprehensive guidelines which include annotation of 28 features for variant classification into five broad categories (i.e., pathogenic, likely pathogenic, benign, likely benign, and VUS) [36]. The variant classification was calculated based on the weighted 28 attributes. For variants, pathogenicity was weighted as very strong (PVS1), strong (PS1-4), moderate (PM1-6), or supporting (PP1-5). Similarly, for benign, it was weighted as stand-alone (BA1), strong (BS1-4), or supporting (BP1-7). The combination of these attributes put together in the algorithm of the genetic variant interpretation tool [37] classifies the variant. The guidelines associated with the weighted attributes have been detailed in Supplementary Data 1**.**

### Statistical significance of pathogenic variants with global population

The statistical differences in the allele frequencies of pathogenic or likely pathogenic variants were estimated between the Indian population of IndiGen dataset with the global population dataset. The global population datasets include Genome Aggregation Database (gnomAD v3), which is the largest database of 71,702 whole-genome–sequenced individuals from eight populations (African, Amish, Ashkenazi Jews, East Asian, Finnish, Non-Finnish, Latino, and South Asian) [28]; the 1000 Genome Project (1000g2015aug_all) was composed of whole genome sequencing of 2504 individuals from five super populations (Africa, America, Europe, East Asia, and South Asia) [29], and ESP6500 (esp6500siv2_all) composed of 6503 whole exome–sequenced dataset of 2203 African–American and 4300 European–American healthy individuals [38]. The statistical significance was tested by Fisher’s exact test; a *P* value of less than 0.05 was considered significant.

### Ancestry haplotyping of a founder mutation

**Fig. 1:**
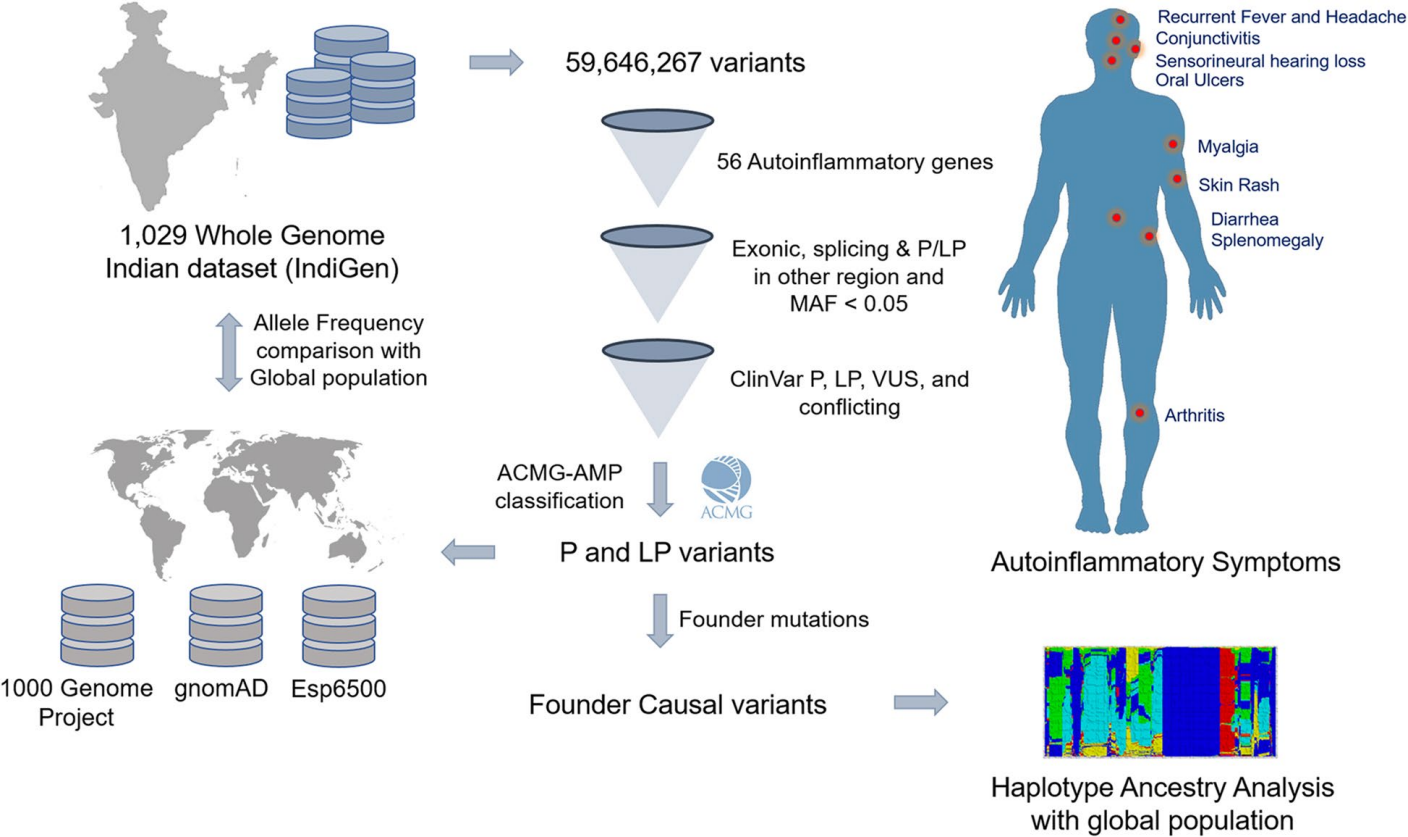
Schematic summarizing the data analysis pipeline utilized in this study

Since several autoinflammatory conditions have geographical associations, we were interested in the pathogenic or likely pathogenic variants which were prevalent and considered as a founder variant in the population. We further explored the haplotype similarity pattern of the founder variant of each individual from the IndiGen dataset to the global population dataset of the 1000 Genome Project. To evaluate the haplotype similarity, we used a tool called fineSTRUCTURE [39] (version 2.1.3) that uses linkage disequilibrium model-based STRUCTURE approach and principal component analysis for population haplotype prediction. Each individual specific chromosomal variant, where founder variants are located, were merged with 2548 individual variants from the 1000 Genome Project. Further, merged variants were pruned by applying a filter of allele frequency of > 1% and allele count > 1000 to get maximum genotype rate using a bespoke bash script. We phased the filtered merged VCF using SHAPEIT v2.r900 tool [40]. Further, the fineSTRUCTURE pipeline was used to analyze the phased VCF and plotted using R script. The overall workflow adopted in this study has been represented in Fig. 1.

## Results

### Genetic variants in the autoinflammatory genes from different datasets

**Fig. 2:**
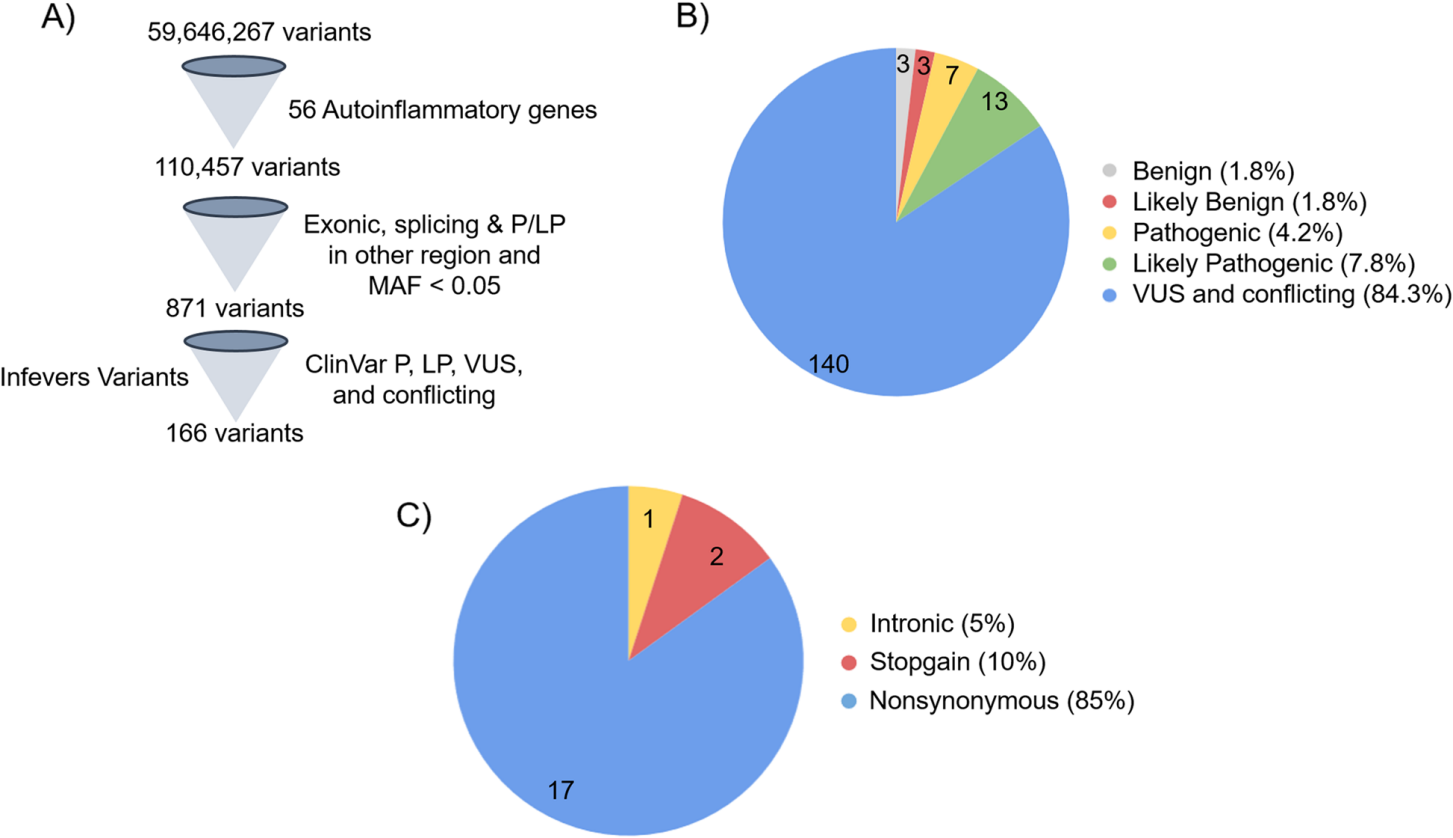
Variant filtering and classification based on ACMG-AMP guidelines. **A** Variant prioritization for ACMG classification. **B** Variant classification based on ACMG-AMP guidelines. **C** Pathogenic and likely pathogenic variants classification based on the type of variant

The IndiGen dataset consisted of a total of 50,517,048 single-nucleotide variants and 5,381,074 InDels, out of which, 110,457 variants were retrieved from 56 genes associated with the 47 autoinflammatory disorders. We further prioritized 871 exonic, splicing and ClinVar pathogenic and likely variants in other regions whose minor allele frequency is less than 0.05. Further, these variants were mapped on the merged ClinVar pathogenic, likely pathogenic, VUS, and conflicting variants and on Infevers variants that retrieved 166 variants for further analysis as shown in Fig. 2A.

### Variant classification based on ACMG and AMP guidelines

These 166 variants were further scrutinized according to the ACMG-AMP guidelines and classified as pathogenic, likely pathogenic, benign, likely benign, and VUS. In our analysis, we found 7 variants as pathogenic and 13 variants as likely pathogenic (Table 2), while 6 variants were benign and likely benign, and 140 variants were VUS or variants with conflicting evidence (Fig. 2B). Out of 20 pathogenic and likely pathogenic variants, there are 1 intronic, 2 stopgain, and 17 nonsynonymous variants (Fig. 2C). The detailed variant annotation and classification are tabulated in Supplementary Table 1.

**Table 2:** Pathogenic and likely pathogenic variants classified by ACMG-AMP guidelines

Gene	Variant	SNPID	Protein change	ACMG criteria	Classification	Inheritance	Disease
ADAR	chr1:154602065:G>C	rs145588689	c.C577G: p.P193A	PM1, PP3, PM3, PP4	Likely pathogenic 5	AR	AGS
IL36RN	chr2:113060943:T:C	rs148755083	c.115+6T>C: p.Arg10ArgfsX1	PS2, PS3, PP5	Pathogenic 2	AR	DITRA
IL36RN	chr2:113062547:C>T	rs144478519	c.C338T: p.S113L	PM1, PP3, PS3, PM3, PP1, PP4	Pathogenic 3b	AR	DITRA
AP1S3	chr2:223777862:A>C	rs116107386	c.T11G: p.F4C	PM1, PP3, PS3, BS4	Likely pathogenic 2	AD	Pustular psoriasis
RNASEH2C	chr11:65720385:G>A	rs78635798	c.C205T: p.R69W	PM1, PM2, PP3, PP5, PS3, PP1-M	Pathogenic 3a	AR	AGS
MVK	chr12:109586107:A>G	rs104895364	c.A613G: p.N205D	PM1, PM2, PP3, PP4	Likely pathogenic 5	AR	HIDS
MVK	chr12:109596515:G>A	rs28934897	c.G1129A: p.V377I	BP4, PS4, PM3, PP1-M	Likely pathogenic 2	AR	HIDS
RNASEH2B	chr13:50945445:G>A	rs75184679	c.G529A: p.A177T	PM1, PP3, PP5, PS3, BS3, PP4	Likely pathogenic 2	AR	AGS
PSTPIP1	chr15:77031192:C:T	rs751668240	c.C850T: p.Q284X	PVS1, PM2, PP3	Pathogneic 1c	AD	PAPA
NOD2	chr16:50711206:C>T	rs2076754	c.C1295T: p.A432V	PM1, PM2, PP3, BS3, PS4	Likely pathogenic 2	AD	Blau Syndrome
NOD2	chr16:50711301:G>T	rs104895492	c.G1390T: p.G464W	PM2, PP3, PS3, PP1	Likely pathogenic 2	AD	Blau Syndrome
NOD2	chr16:50712048:C>T	rs104895440	c.C2137T: p.R713C	PM2, PP3, PS3	Likely pathogenic 2	AD	Blau Syndrome
CARD14	chr17:80184015:G>A	rs200731780	c.G452A: p.R151Q	PM2, BP4, PS3	Likely pathogenic 2	AD	CAMPS
CARD14	chr17:80198685:C:T	rs200379060	c.C1234T: p.R412X	PVS1, PM2	Likely pathogenic 1	AD	CAMPS
NLRP7	chr19:54936400:G>A	rs104895525	c.C2161T: p.R721W	PM2, PP3, PS1	Likely pathogenic 2	AR	HYDM1
NLRP12	chr19:53810605:G:A	rs199881207	c.C1054T: p.R352C	PM1, PM2, PS3, PP3	Likely pathogenic 2	AD	FACS
ADA2	chr22:17203564:G>A	rs148936893	c.C626T: p.P209L	PM1, PM2, BP4, PP5, PS3, PM3, PP1	Pathogenic 3a	AR	DADA2
ADA2	chr22:17209538:C>A	rs200930463	c.G140T: p.G47V	PM1, PM2, PP3, PP5, PM5	Likely pathogenic 4	AR	DADA2
ADA2	chr22:17209539:C>G	rs202134424-G	c.G139C: p.G47R	PM1, PM2, PP3, PS1, PS3, PS4	Pathogenic 2	AR	DADA2
ADA2	chr22:17209539:C>T	rs202134424-T	c.G139A: p.G47R	PM1, PM2, PP3, PP5, PS3, PS4	Pathogenic 2	AR	DADA2

*AGS* Aicardi-Goutières syndrome, *DITRA* deficiency of interleukin-36-receptor antagonist, *HIDS* hyper IgD syndrome, *CAMPS* CARD14-mediated pustular psoriasis, *HYDM1* hydatidiform mole, recurrent, 1, *DADA2* deficiency of adenosine deaminase 2, *AR* autosomal recessive, *AD* autosomal dominant

### Comparison of variant frequency with the global population

Allele frequencies of 20 pathogenic or likely pathogenic variants were compared with the global populations that include gnomAD V3, 1000 Genome Project (1000g2015aug_all), and ESP6500 (Esp6500siv2_all). We found 17 out of 20 variants were significantly different from the Indian population compared with the global populations (Fisher’s exact *P* < 0.05). All the 17 variants were significantly different in comparison with the gnomAD database or its subpopulations, while 3 variants had significantly different allele frequencies compared with the 1000 Genomes dataset and 1 variant compared with the Esp6500 European exome dataset. We could not perform Fisher’s exact test for 12 variants of the 1000 Genome Project and Esp6500 each as the allele frequencies were unavailable in the respective datasets.

**Fig. 3:**
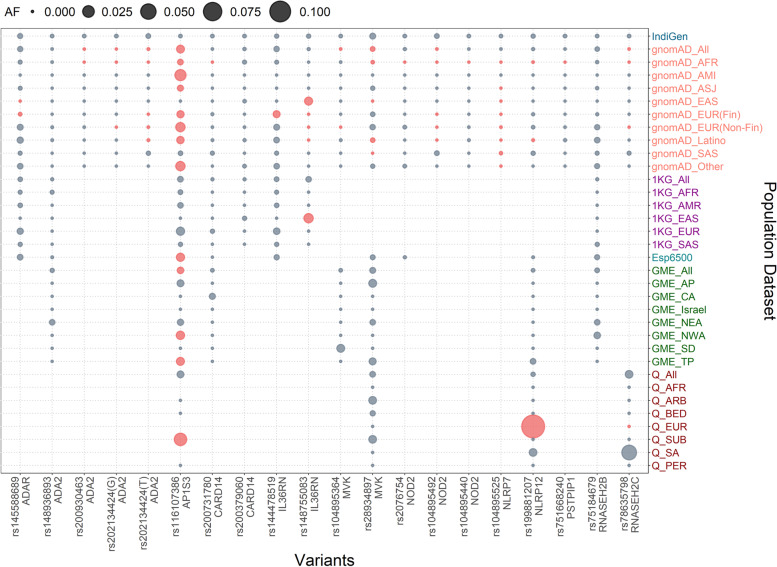
Comparison of allele frequency of pathogenic and likely pathogenic variants in the IndiGen population with the gnomAD, 1000 genome project (1KG), Esp6500, Greater Middle East (GME), and Qatar (Q) and its subpopulation: Amish (AMI), European (Finnish) (EUR (Fin)),, European (Non Finnish) (EUR (Non Fin)), African (AFR), Ashkenazi Jewish (ASJ), East Asian (EAS), South Asian (SAS), American (AMR), Bedouin (BED), Sub-Saharan African (SAF), European (EUR), South Asian (SOU), African Pygmy (APY), Arab (ARA), Persian (PER), Northwest Africa (NWA), Northeast Africa (NEA), Turkish Peninsula (TP), Syrian Desert (SD), Arabian Peninsula (AP), and Persia and Pakistan (PP). *CA*, Central Asia. Significant allele frequency highlighted with the red circle

In this analysis, two pathogenic variants rs144478519 and rs148755083 in the *IL36RN* gene each had 0.1% allele frequency in the Indian population (IndiGen) that were significantly less in comparison with the European population and East Asian population respectively in both 1000 Genome Project and gnomAD. Interestingly, the latter variant, rs148755083, is private to the East Asian population. Similarly, a pathogenic variant rs78635798 in *the RNASEH2C* gene had 0.05% allele frequency in the IndiGen dataset and was present only in the South Asian population. A likely pathogenic variant rs104895492 in the *NOD2* gene with high allele frequency in Indian and South Asian population dataset of 0.15 and 0.16%, while absent in other populations. Another likely pathogenic variant rs116107386 in the *AP1S3* gene whose allele frequency is significantly low in the Indian population (i.e., 0.05% in comparison with the four populations of the gnomAD that includes Amish, European (Finnish and Non-Finnish), and Latino as well as European population of the 1000 Genome Project and ESP6500). An interesting likely pathogenic variant rs28934897 in the *MVK* gene also popularly known as Dutch mutation has high allele frequency in the Indian population (i.e., 0.34%, while absent in the South Asian populations of the control population database). The allele frequency, allele count, and allele number of all the pathogenic variants with a comparison with the gnomAD database and its population have been summarized in Table 3. The comparison with all three global populations 1000 Genome Project, gnomAD, and ESP6500 and also with Greater Middle East and Qatar populations *27,408,750* has been tabulated in Supplementary Table 2 and well represented in Fig. 3.

**Table 3:**
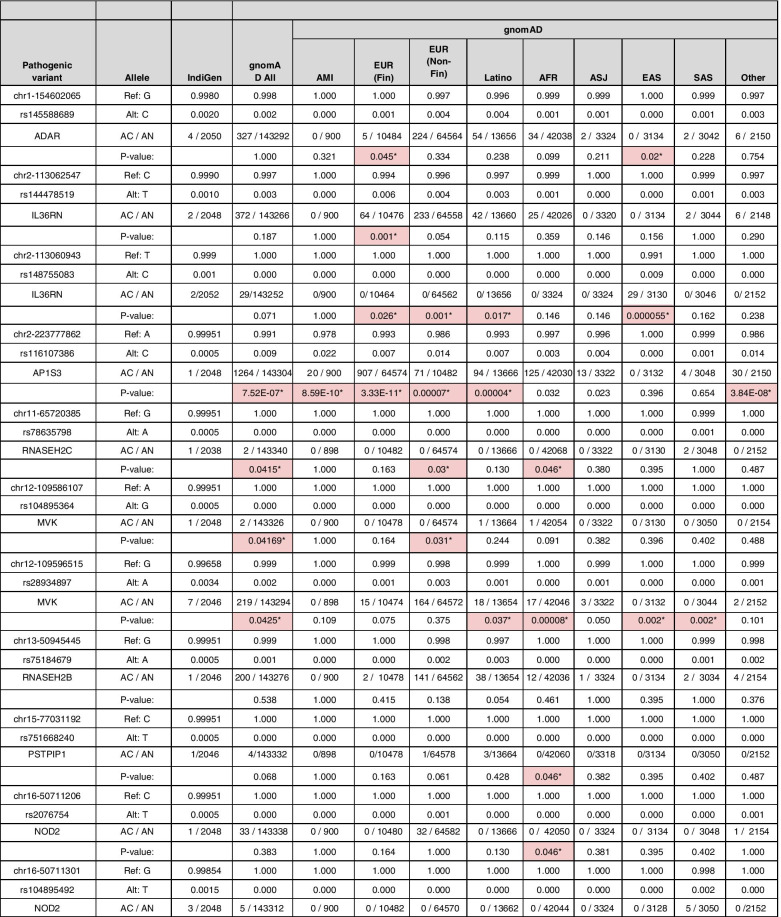

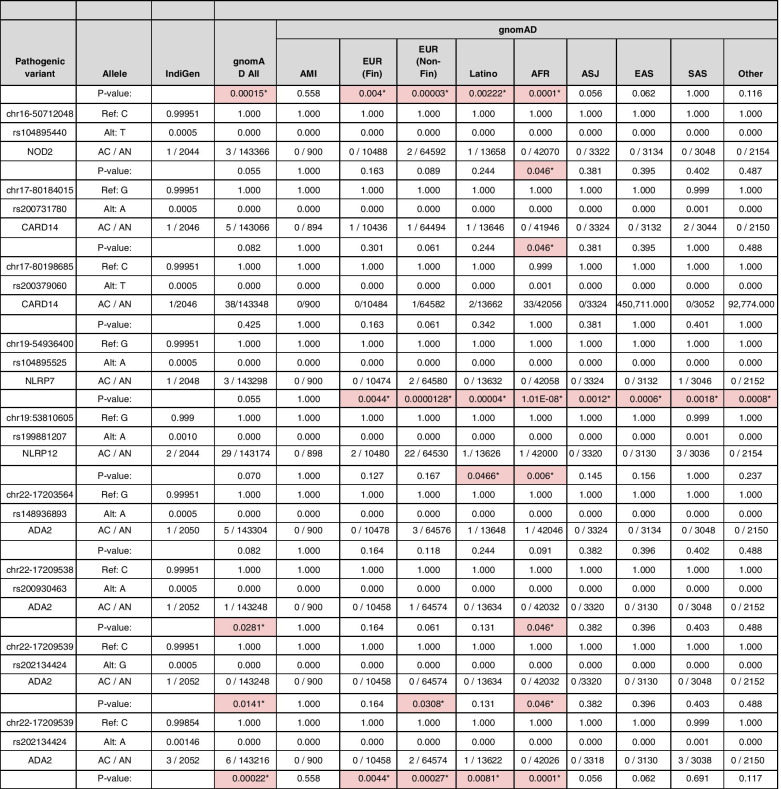
IndiGen allele frequency comparison *P* value (< 0.05) of autoinflammatory disorder pathogenic and likely pathogenic variants with the gnomAD (global) database

*AMI* Amish, *EUR* (*Fin*) European (Finnish), *EUR* (*Non-Fin*) European (Non-Finnish), *AFR* African, *ASJ* Ashkenazi Jewish, *EAS* East Asian, *SAS* South Asian

Significant values are marked with asterisk (*) and cells colored in red

### Ancestry haplotyping

**Fig. 4:**
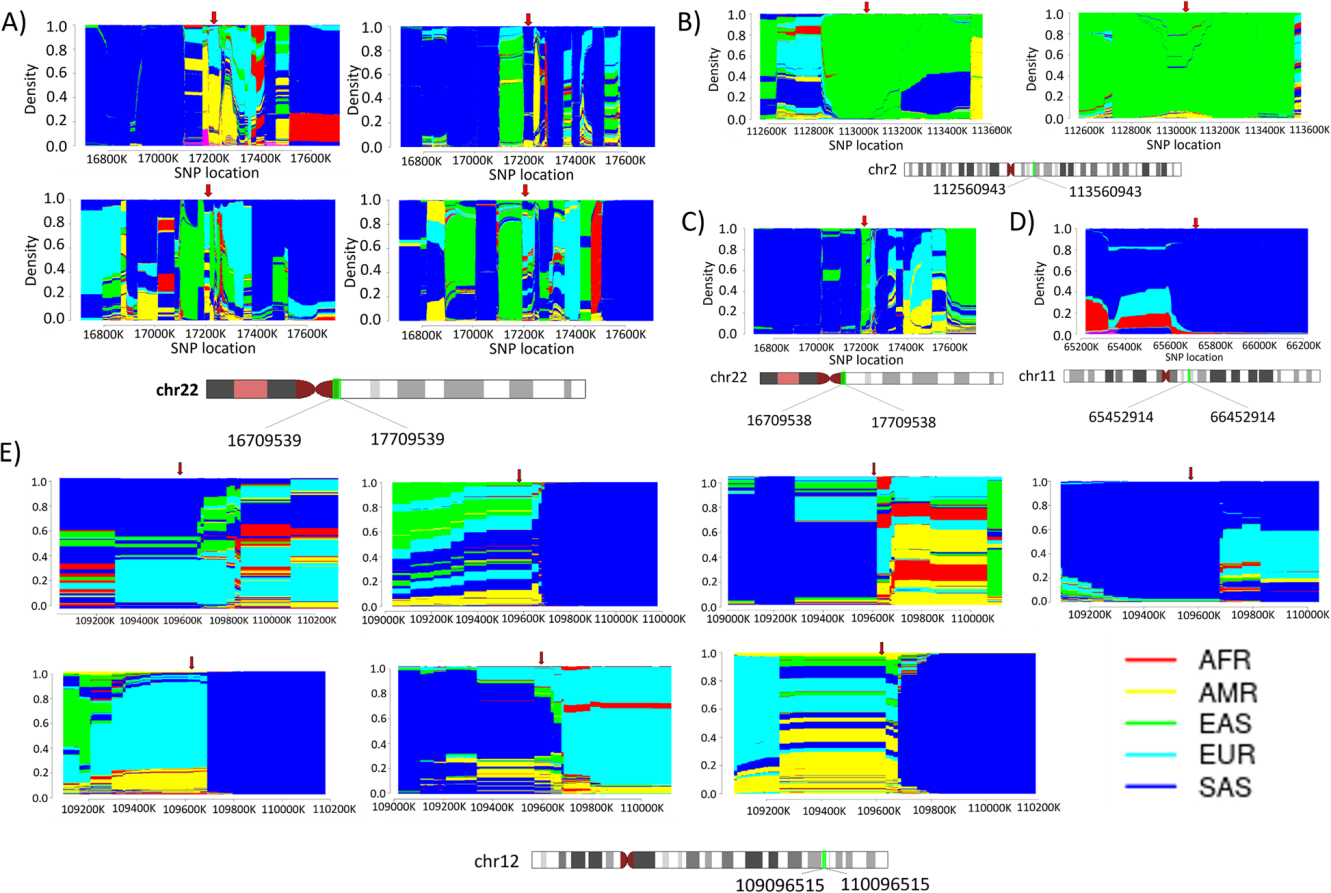
Visualization of haplotype ancestry of founder variants in IndiGen individuals with 5MB upstream and downstream mapped to the five superpopulation of 1000 Genome Project **A**) The chromosomal painting of Georgian Jewish founder variant rs202134424-T (marked with red arrow) in four IndiGen individuals. **B**) The chromosomal painting of East Asian founder variant rs148755083 (marked with red arrow) in two IndiGen individuals. **C**) The chromosomal painting of Georgian Jewish founder variant rs202134424-G found in a single IndiGen individual. **D**) The chromosomal painting of Asian founder variant rs78635798 (marked with red arrow) in a single IndiGen individual. **E**) The chromosomal painting of Dutch population founder variant rs28934897 in seven IndiGen individuals. *AFR* Africa, *AMR* America, *EAS* East Asia, *EUR* European, *SAS* South Asian

We found six causal founder variants (i.e., rs28934897, rs148755083, rs202134424-T, rs202134424-G, rs200930463, and rs78635798 in the *MVK, IL36RN, ADA2, ADA2, ADA2,* and *RNASEH2C* genes, respectively). The first three variants rs28934897, rs202134424-T, and rs148755083 were present in seven, four, and two individuals, respectively while other three variants (rs202134424-G, rs200930463, and rs78635798) were found in a single individual of the IndiGen dataset in heterozygous state. To identify the haplotype ancestry, we have applied filters of allele frequency > 1% on the merged IndiGen individual variant with the 1000 Genome Project that results on an average of 23,843, 784,354, 137,671, 137,701, 137,783, and 473,514 variants with approximately 99% genotype rate for variants rs28934897, rs148755083, rs202134424-T, rs202134424-G, rs200930463, and rs78635798, respectively. After performing chromosomal painting using fineSTRUCTURE, we identified six out of seven individuals had admixed European haplotype around the rs28934897 variant along with South Asian in four individuals and American haplotype in two individuals, while one individual had a complete South Asian haplotype. Similarly, those  with variant rs202134424-G/T had admixed European haplotype in three individuals, out of which two had admixed American and one had admixed South Asian haplotype, while one individual had complete South Asian haplotype. Interestingly, a variant rs148755083 identified in two individuals in IndiGen was found to have East Asian haplotype. Also, the variant rs200930463 harbored by an IndiGen individual was found to have East Asian haplotype. While an individual harboring a variant rs78635798 had a South Asian haplotype around the variant. The painted chromosomal region 500 KB upstream and downstream of the founder variant has been represented in Fig. 4.

## Discussion

Autoinflammatory disorders are a group of Mendelian disorders caused by genetic defects in the genes involved in the regulation of innate immune systems. There are more than 50 genes that are associated with the autoinflammatory disorders as curated by the experts of the International Union of Immunological Societies (IUIS) [33] and Infevers [8, 34]. There are a number of genetic epidemiological studies that have been carried out around the world which suggest high prevalence of the distinct autoinflammatory disorders in different regions of the world [41–46]. The most common autoinflammatory disorder is familial Mediterranean fever (FMF) found to be very common in Middle Eastern countries [42, 44, 45]. A study performed on 1299 Armenian patients affected with FMF found a high likelihood of carrier individuals with disease manifestation [45]. Another study revealed the carrier with high frequency of *MEFV* mutation (i.e., 1 in 5 in healthy Armenian individuals) [44]. A comprehensive study performed by our group on more than 2000 whole exome sequence dataset of the Mediterranean region revealed a carrier frequency of 8% in the population [41]. The 8-year epidemiological retrospective study for the cryopyrin-associated periodic syndrome (CAPS) caused due to the *NLRP3* gene estimated 1/360,000 prevalence in France [43]. Another study performed by Houten et al. estimated 1:65 carrier frequency of *MVK* mutation in the Dutch population associated with hyper IgD syndrome [46]. While only a handful of genetically characterized autoinflammatory diseases have been reported in India [22, 47] including from our group [48], the genetic frequencies of variants in the population remain an enigma. The recent availability of genome-wide data for the cosmopolitan Indian population has motivated us to understand the genetic epidemiology of autoinflammatory diseases in India. Our systematic analysis of variants and reclassification according to the ACMG & AMP guidelines revealed a total of 20 genetic variants which could be classified as pathogenic or likely pathogenic.

In total, 36 genomes in the IndiGen dataset had at least one pathogenic/likely pathogenic variant. Of the individuals with carriers for any of the pathogenic/ likely pathogenic variants, in the *ADA2* genes, causing deficiency of adenosine deaminase 2 (DADA2) had the maximum number of 4 unique variants in 6 individuals. The second maximum number of variants that was present in the *NOD2* gene that causes Blau syndrome and had 3 unique variants in 5 individuals. This was followed by the *MVK, NLRP12,* and *IL36RN* genes causing hyper IgD syndrome, (HIDS) and generalized pustular psoriasis (GPP) with 2 unique variants each, in 8 and 4 individuals, respectively. A total of 4 and 2 individuals were carriers for a causal variant in the *ADAR* and *NLRP12* gene causative for Aicardi-Goutières syndrome (AGS) and familial cold autoinflammatory syndrome (FACS). While the remaining 7 genes (*AP1S3, RNASEH2C, RNASEH2B, CARD14, NLRP7, PSTPIP1,* and *CARD14*) have only a single individual carrier for the causal variant.

Out of 20 causal variants, 7 were pathogenic (1 intronic, 1 stopgain, and 5 nonsynonymous), and 13 are likely pathogenic variants (1 stopgain and 12 nonsynonymous) by ACMG-AMP guidelines. We identified two pathogenic variants in the *IL36RN* gene that includes a nonsynonymous variant (c.338C>T: p.S113L) rs144478519 and intronic variant rs148755083 (c.115+6T>C: p.Arg10ArgfsX1). A nonsynonymous variant rs144478519 was found in multiple unrelated patients of different ancestries affected with GPP in the homozygous or compound heterozygous state. This variant falls in a region that is evolutionarily conserved with proximity to the binding site involved in receptor interaction. This interaction is responsible for the IL-36 signaling system for the inhibition of the activity of interleukin-36 that further inhibits the local autoinflammatory response [23]. Functional studies also revealed a significant increase in proinflammatory cytokines [49–51]. The latter intronic variant rs148755083 is predominantly found in the East Asian populations with allele frequency of ~ 1% in the global dataset. This variant has been found to be highly prevalent in patients of Japanese and Chinese ancestry and was identified as founder mutation in both the population [50, 52–54]. A nonsynonymous pathogenic variant rs78635798 (c.205C>T: p.R69W) in *the RNASEH2C* gene causative of AGS had very low allele frequency in the global populations (i.e., < 0.01%) and falls in the functionally important domain. Functional studies revealed *RNASEH2C* p.R69W had a significant reduction in the thermal stability of RNase H2 complex [55]. This variant has been recurrent and considered as a founder mutation in the Asian population as well; it segregates with the disease in the family [56]. Other founder pathogenic variants from the Georgian Jewish population (p.G47R) rs200930463-T and rs200930463-G in *the ADA2* gene cause DADA2 with the carrier frequency in this population as 10.2% with the high prevalence of the disease [57]. However, in the global population datasets, it has a very low allele frequency of < 0.0002%. The functional analysis showed a marked reduction of ADA2 activity in comparison with the wild type in the homozygous state [57, 58]. Recently, a case series of 33 DADA2-affected patients from India has been reported and found this variant p.G47R to be prevalent in the Jain/Aggarwal community [59]. Another *ADA2* variant at the same amino acid position of the ADA2 protein (p.G47V) rs200930463 was in a trans compound heterozygous state with p.W246S in the patient affected with DADA2 [57]. Also, functional studies revealed the complete absence of ADA2 protein in cells transfected with p.G47V and p.W246S as well as lower amounts in drosophila S2 cells [57]. The pathogenic nonsynonymous variant rs148936893 (c.C752T: p.P251L) in *the ADA2* gene associated with the DADA2 has a low frequency of < 0.02% in the global population datasets. This variant was found to be segregated with the disease in a German family in a trans compound heterozygous state. Functional studies revealed ADA2 activity has been severely compromised and also indicated intracellular elevated levels of ADA2 protein [57].

Another founder mutation of Dutch ancestry (c.G1129A: p.V377I) rs28934897 in the *MVK* gene causes HIDS with very high carrier frequency (i.e., 1:65) [46]. This variant has been reported in multiple patients with HIDS of different ancestry and found variant segregation with the disease in the family either in compound heterozygous or homozygous state [60–62]. In vivo and in vitro functional studies have revealed a significant decrease in the enzymatic activity of MVK protein [63]. Recently, we have found a p.V377I variant in our six patients of South India ancestry in a trans compound heterozygous state residing in the same region and identified as founder in the South Indian population [48]. Another likely pathogenic *MVK* variant rs104895364 (c.613A>G: p.N205D) was found in a trans compound heterozygous state in two patients affected with HIDS of the same family [48]. It has a very low allele frequency of < 0.005% in the global population datasets (i.e., 1000 Genome Project, gnomAD, and Esp6500). The variant falls in the functionally important domain and was found in multiple patients affected with HIDS of different ancestry [64–67]. A likely pathogenic nonsynonymous variant rs145588689 (c.577C>G: p.P193A) in *the ADAR* gene associated with Aicardi-Goutières syndrome (AGS) was found to be segregated with disease in compound heterozygous state in 22 out of 23 unrelated families as well as in multiple unrelated AGS patients [68, 69]. This variant falls in the functional domain that disrupts the interaction between Z-DNA/Z-RNA binding thus upregulating IFN-stimulated genes [70]. Another likely pathogenic variant rs2076754 (c.C1295T: p.A432V) in *the NOD2* gene associated with Blau syndrome/ Crohn’s disease does not have any significant difference in comparison with the wild type [71]. However, it has a low allele frequency of ≤ 0.02% in the global population dataset, and also the odds of occurring in Crohn’s disease patients is significantly higher than the controls [72]. Another nonsynonymous likely pathogenic mutation (c.G1390T: p.G464W) rs104895492 in the *NOD2* gene has a low allele frequency of 0.003% in the global population. A reporter assay revealed this mutation to cause hyperactivity of NOD2-mediated NF-KB signaling in the absence of ligands [73]. The variant segregates with the disease in the mother and daughter, both were affected with Blau syndrome [74]. Another likely pathogenic nonsynonymous variant rs104895440 (c.C2137T: p.R713C) in the *NOD2* gene has very low allele frequency of < 0.003% in the global population datasets. In vitro functional studies of the variant revealed major impairment of the peptidoglycan-induced response [71]. A nonsynonymous mutation (c.C1054T: p.R352C) in the *NLRP12* gene found in two patients suffering from familial cold autoinflammatory syndrome (FCAS) of different ancestries. Functional study of the mutation revealed the increase in the speck formation as well as activation of the caspase 1 signaling in comparison with the wild type [11]. A likely pathogenic nonsynonymous variant rs200731780 (c.452G>A: p.R151Q) in the *CARD14* gene associated with CARD14-mediated pustular psoriasis (CAMPS) that has been predicted as benign by computational prediction tools. However, in vitro functional studies of p.R151Q have shown a significant increase (i.e., 18 folds in the NF-KB activation that leads to CAMPS) [75, 76]. It has a very low allele frequency of < 0.03% in the global population datasets (i.e., 1000 Genome Project, gnomAD, and Esp6500). Another likely pathogenic variant rs116107386 (c.11T>G: p.F4C) in the *AP1S3* gene associated with pustular psoriasis has a frequency of ~ 1% in the global population. However, in transfection studies in HEK293 cells, it showed a significant decrease of the mutant protein in comparison with the wild type that leads to marked inhibition of downstream signaling. The allele frequency was also found to be significantly higher in the patient than controls and falls in functionally important domains [77].

In this study, we have prioritized six causal founder variants. This includes rs28934897, rs148755083, rs202134424-T, rs202134424-G, rs200930463, and rs78635798 in the *MVK*, *IL36RN*, *ADA2*, *ADA2*, *ADA2*, and *RNASEH2C* genes, respectively. The variant (rs28934897) in the *MVK* gene is harbored in seven individuals of the IndiGen dataset in heterozygous state. On performing haplotype ancestry analysis, we found six out of seven individuals had admixed European ancestry. The occurrence of the European haplotype at the founder variant in the Indian population could be due to the invasion or migration of the Europeans in India [78]. The founder Dutch mutation p.V377I (rs28934897) along with the splicing mutation c.226+2delT in the *MVK* gene in trans compound heterozygous state was found to be more common in the Indian population than reported [48]. An intronic variant c.115+6T>C (rs148755083) in the gene *IL36RN* implicated in GPP was predominantly identified and considered as a founder variant in the East Asian population [50, 52–54]. We have identified this variant in two IndiGen individuals belonging to the Eastern part of India and was found to have East Asian ancestry. Since it has high frequency in the Eastern parts of India, variants could be screened, and the government could take proactive measures. We have also identified three variants p.G47V (rs202134424-T), p.G47V (rs202134424-G), and G47R (rs200930463) in the *ADA2* gene implicated in DADA2 known to be founder with high frequency in the Georgian Jewish population [57]. In IndiGen, out of the five individuals harboring these variants, three of them had European admixture, one East Asian, and one South Asian ancestry. A recent study performed in Indian DADA2 patients identified p.G47R as a founder variant in the Aggarwal/Jain community [59]. The Aggarwal community mainly resides in North India and is a descendant of the Indo-European migrants that had high frequency of the ADA2 causal variant [59, 79]. The *RNASEH2C* variant p.R69W (rs78635798) implicated in AGS found in a single individual of IndiGen had South Asian haplotype ancestry. This variant was considered as a founder variant in the Asian populations [56].

In contrast with similar approaches towards understanding the genetic epidemiology of autoinflammatory diseases in other populations, three of the causal variants in the present analysis overlapped with our previous analysis of the Middle Eastern population [21]. Compared to the variant frequencies in the global populations, a number of disease alleles have frequencies in Indian population higher than global datasets including rs116107386, rs78635798, rs104895364, rs28934897, rs104895492, rs200930463, and rs202134424. Similarly, a number of variants have allele frequencies less than global populations like rs148755083 in *IL36RN* for pustular psoriasis. We surmise that the allele frequencies also correlate with the frequency of variants in clinical settings. For example, a recent case series of HIDS from our group suggests the clinical and genetic characteristics of patients. Incidentally, the prevalent variant rs148755083 with a founder effect was also the frequency variant identified in the case series [48].

The study has many caveats, the major being the dataset encompasses only a limited sample of cosmopolitan Indians, and therefore might not adequately cover smaller endogamous and ethnic groups. Secondly, the annotation of variants based on evidence from already proven cases, and therefore preclude novel and potentially pathogenic genetic variants.

## Conclusions

The present analysis of genomes suggests that a number of autoinflammatory disease variants are prevalent in India. A subset of the variants were founders and were mainly descendants of different ancestry (i.e., European and East Asian due to migration or invasion in India). The causal founder autoinflammatory variant had high frequency with respect to their geographical regions or community. That could be considered a hotspot variant for the distinct population. The respective government could undertake the initiative and could perform the low-cost population screening so that it could provide better health care facilities to the population.

## Supplementary Information

**Additional file 1: Supplementary Data 1.** Detailed description of 28 ACMG-AMP guidelines for variant classification.

**Additional file 2: Supplementary Table 1.** Autoinflammatory variants annotation and their classification according to the ACMG-AMP guidelines.

**Additional file 3: Supplementary Table 2.** IndiGen allele frequency comparison *p*-value (<0.05) of autoinflammatory disorder pathogenic and likely pathogenic variants with the global databases included gnomAD V3, 1000 Genome Project, Esp6500, GME, and Qatar with their subpopulation. AMI: Amish, EUR (Fin): European (Finnish), EUR (Non Fin): European (Non Finnish), AFR: African, ASJ: Ashkenazi Jewish, EAS: East Asian, SAS: South Asian, AMR: American, BED Bedouin, SAF Sub-Saharan African, EUR European, SOU South Asian, APY African Pygmy, ARA ARAB, PER Persian, NWA: Northwest Africa, NEA: Northeast Africa, TP: Turkish Peninsula, SD: Syrian Desert, AP:Arabian Peninsula, and PP: Persia and Pakistan, NA not applicable . Significant values are marked with * and cells colored in red.

## Data Availability

All data generated or analyzed during this study are included in this published article (and its Supplementary information files).

## Abbreviations

VUS: Variant of uncertain significance
ACMG-AMP: American College of Medical Genetics and Genomics and Association of Molecular Pathology
FMF: Familial Mediterranean fever
CAPS: Cryopyrin-associated periodic syndrome
GME: Greater Middle East
IUIS: International Union of Immunological Societies
DADA2: Deficiency of adenosine deaminase 2
HIDS: Hyper IgD syndrome
GPP: Generalized pustular psoriasis
AGS: Aicardi-Goutières syndrome
FCAS: Familial cold autoinflammatory syndrome
CAMPS: CARD14-mediated pustular psoriasis
