# Supplementary material for: Genetic epidemiology of autoinflammatory disease variants in Indian population from 1029 whole genomes
Source: J Genet Eng Biotechnol. 2021 Dec 14;19:183. doi: 10.1186/s43141-021-00268-2 (PMC8671593; doi:10.1186/s43141-021-00268-2)
Supplement: Supplementary file 1 — Additional file 1: Supplementary Data 1. Detailed description of 28 ACMG-AMP guidelines for variant classification. [file 43141_2021_268_MOESM1_ESM.docx]

**Supplementary Data 1. A detailed description of the methodology adopted for the annotation of ACMG-AMP guidelines.**

**Determination of PVS1**

We determine PVS1 for variants annotated as nonsense, frameshift, and non-canonical splicing using ANNOVAR. We checked whether the variant falls were in the last exon or last 50 base pair of the penultimate exon of the transcript by making bed files of nonsense, frameshift, and non-canonical splicing variants and overlapped with the last exon and last 50 bp of the penultimate exon. At last, we checked whether loss of function (LOF) is the main cause of disease for that gene.

**Determination of PS1 and PM5**

For determining PS1 and PM5 we matched the amino acid change of the nonsynonymous variants with the pathogenic and likely pathogenic variants of ClinVar. If the pathogenic or likely pathogenic variant in clinvar had the same amino acid change as well position at the protein level but differed at the genomic level, they were marked as PS1. While for the novel nonsynonymous variants, which had the same amino acid position at the protein level but changes in the amino acid can be different were marked as PM5.

**Determination of PS2 and PM6**

We performed extensive literature mining for the variant to evaluate the de-novo mode of inheritance for the variant. We analyze the pedigree and literature to find the origin of a new mutation in the offspring which were absent in the parents then it was marked as PS2. In case, the pedigree or the information was not sufficiently confirming the identity of the parents but was hypothetically de-novo then it was regarded as PM6.

**Determination of PS3 and BS3**

These attributes were annotated by literature screening to evaluate the variants for which in vivo and in vitro functional studies have been performed. If in the studies, a variant was reported to be causative in the disease, it was marked as PS3. While a benign effect of the variant was marked as BS3.

**Determination of PS4**

This attribute was also determined by the literature screening of case-control studies to determine the Odds Ratio (OR). If the OR was found to be greater than 5 and the Confidence interval (CI) was more than 1, then the variant was reported as PS4.

**Determination of PP1, PP1-M, PP1-S, and BS4**

We performed literature screening to analyze if the variants co-segregation in the family with the disease. If the variant was present in all affected individuals in a family, then it is marked as PP1. The PP1 has different variations depending on the number of individuals the variant has segregated with the disease i.e. strong (PP1-S), moderate PP1-M), and supporting (PP1). If the variant was absent in any of the members of an affected family member or present in an unaffected member of the family, then due to lack of segregation it was marked as BS4.

**Determination of PP2 and BP1**

We calculated the total number of pathogenic missense and stop gain variants for each gene using Clinvar. if the percentage of missense variant was >80% and stop-gain variant was <20% then for the nonsynonymous variant was marked as PP2. Otherwise, if the stop-gain variant was more than 80% and the nonsynonymous variant was < 20%, then the nonsynonymous variant was marked as BP1.

**Determination of PP3 and BP4**

The variants were annotated using the ljb26_all database of the ANNOVAR annotation tool to determine the PP3 and BP4 parameters. The variants were scored using the SIFT and PolyPhen2, two of the popularly used in-silico callers to score the variants for their predicted pathogenicity. While SIFT cut-offs classify the variants as deleterious or tolerated, PolyPhen2 predictions classify them into 3 categories as probably damaging, Possibly Damaging, or Benign. We also considered CADD scores which contain PHRED scaled scores, if the score is 10 predicted, the 10% most deleterious variants, similarly a score of 20 predicts 1% the most deleterious variant. However, we rationally considered the CADD scores above 15 to be interpreted as deleterious in our analysis. The variants classified as deleterious/damaging by at least two of the three in-silico callers were marked as PP3 (pathogenic). Similarly, variants were marked as BP4 if the majority of the in-silico tools predicted them to be benign/tolerated.

**Determination of PP4**

We used the OMIM database which is a comprehensive database of human genes and genetic phenotype whose main focus is the relationship of phenotype and genotype in Mendelian disorder and 15,000 genes. It is used to determine whether the disease was associated with just a single gene etiology. The variant was consistent and inherited in the family. The total number of benign should be less than 50% in a gene. All the variants fulfilling these criteria were marked as PP4.

**Determination of PP5 and BP6**

Variants were marked as PP5 and BP6 using publicly available database ClinVar (version 2020-01-13). Those variants which had non-conflicting pathogenic/likely pathogenic calls from reputable laboratories were marked as PP5. Similarly, variants having non-conflicting benign/likely benign calls were marked as BP6.

**Determination of PM1**

We took the protein domains and their corresponding coordinates from the Pfam in the UCSC gene track present in the UCSC browser and intersected the variant coordinates upon them. If the mutation hotspot was within these domains, then the variant was marked as PM1.

**Determination of PM2, BA1, BS1, and BS2**

Control population datasets i.e. 1000 Genome Project (ALL.sites.2015_08), Exome Sequencing Project (esp6500siv2_all), Exome Aggregation Consortium (exac03) and gnomAD V3 derived from ANNOVAR tool databases were used to annotate the variants as BA1, BS1, and PM2. All those variants which had MAF more than 5% in any of the four population datasets were marked as BA1 whilst variants having MAF between 1 and 5% were considered as strong evidence to be benign for Mendelian disorder were marked as BS1. In case the variant is absent from all of the control population datasets or is at extremely low frequency in autosomal recessive i.e. < 0.05%, it was classified as moderate evidence to be pathogenic (PM2). All variants that occurred in genes at greater than 1% frequency in the IndiGen dataset, regardless of population allele frequency, were marked as BS2.

**Determination of PM3 and BP2**

We first determined the mode of inheritance using OMIM. Using literature screening, we checked whether there were two heterozygous variants and the disorder was autosomal recessive. If both the mutations were found to be in trans they were marked as PM3. Similarly, if these were in cis they were considered to be BP2.

**Determination of PM4 and BP3**

For annotating in-frame insertions or deletions by overlapping them with the repeated region in the human genome using repeat masker. If the in-frame insertions or deletions fall in the repeated they were marked as BP3, otherwise, they were marked as PM4

**Determination of BP7**

Synonymous variant not in splice site was marked as BP7.
